# Epoxy–PCM Composites with Nanocarbons or Multidimensional Boron Nitride as Heat Flow Enhancers

**DOI:** 10.3390/molecules24101883

**Published:** 2019-05-16

**Authors:** Richa Agrawal, Joshua Hanna, I. Emre Gunduz, Claudia C. Luhrs

**Affiliations:** Department of Mechanical and Aerospace Engineering, Naval Postgraduate School, Monterey, CA 93943, USA; joshua.hanna@nps.edu (J.H.); emre.gunduz@nps.edu (I.E.G.)

**Keywords:** phase change material, thermal energy storage, epoxy composites, nanomaterials, materials for temperature regulation, paraffin, boron nitride, carbon

## Abstract

The need for affordable systems that are capable of regulating the temperature of living or storage spaces has increased the interest in exploring phase change materials (PCMs) for latent heat thermal energy storage (LHTES). This study investigates n-nonadecane (C_19_H_40_) and n-eicosane (C_20_H_42_) as alkane hydrocarbons/paraffins for LHTES applications. An epoxy resin is used as the support matrix medium to mitigate paraffin leakage, and a thickening agent is utilized to suppress phase separation during the curing process. In order to enhance the thermal conductivity of the epoxy–paraffin composite, conductive agents including carbon nanofibers (CNFs), carbon nanotubes (CNTs), boron nitride (BN) microparticles, or boron nitride nanotubes (BNNTs) are incorporated in different gravimetric ratios. Enhancements in latent heat, thermal conductivity, and heat transfer are realized with the addition of the thermal fillers. The sample composition with 10 wt.% BN shows excellent reversibility upon extended heating–cooling cycles and adequate viscosity for template casting as well as direct three-dimensional (3D) printing on fabrics, demonstrating the feasibility for facile integration onto liners/containers for thermal regulation purposes.

## 1. Introduction

Due to the rapid industrial growth and ever-increasing energy demands, there is an urgent need for the utilization of renewable energy sources for sustainable and environmentally friendly development. Increasing demands for thermal comfort have led to the higher energy consumption of heating, ventilation, and air conditioning (HVAC) systems [1]. Energy saving for such purposes can be attained by the use of thermal energy storage (TES) systems, where the excess thermal energy can be stored to bridge the gap between the energy demands and generation [1,2,3]. For instance, solar thermal energy can be stored during day hours and utilized during off-peak hours/night times with the use of TES systems [4], which are of particular interest for adaptable systems such as removable attachment/liners for portable accommodations or spaces. TES can be subdivided into sensible heat storage (SHS), latent heat storage (LHS), and thermochemical categories. While SHS is the most commonly used method, LHS is considered very promising due to the wide range of available phase change materials (PCMs) with higher thermal storage density, and almost isothermal operation during thermal release and absorption [4].

PCMs are substances that absorb/release thermal energy during a phase transformation, which is typically melting/solidification, and can be categorized into organic, inorganic, and eutectics [4]. Among organic PCMs, paraffins or alkanes with a chemical formula of C_n_H_2n+2_ (*n* = 12–50) have been widely investigated [4,5,6,7,8,9,10,11] due to their chemical stability, high latent heat of fusion, low cost, compatibility with metal containers, and non-corrosive nature [4]. Despite the aforementioned advantages of paraffins, one of their major disadvantages is their low thermal conductivity, which can significantly interfere with their charging/discharging rates [12]. To address the low thermal conductivity of the paraffins, the addition of thermally conducting agents to form paraffin composites has been widely explored [5,6,11,12,13,14]. Conductive fillers including metals [2], metal foams [15], β-AlN [16], and Al [17] have been added to PCMs for thermal conductivity augmentation. However, metallic additives add significant weight and cost to the storage systems [18,19]. More recently, porous carbon materials have been investigated as heat transfer enhancers for PCMs owing to their high thermal conductivities and low densities [18,19,20,21,22,23]. Among carbon allotropes, carbon nanofibers (CNFs) and carbon nanotubes (CNTs) are especially attractive, as their high aspect ratios offer significant enhancement in thermal conductivity as predicted by the effective medium theory [19,24]. Cui et al. [23] added CNFs in different mass ratios to soy wax PCMs and reported superior transient temperature responses from CNF/PCM mixtures relative to standalone PCMs. Wang et al. [25] synthesized paraffin wax and multi-walled carbon nanotube (MWCNT) composites and reported enhancement in the thermal conductivities of both the liquid and solid states with the increasing mass fraction of MWCNTs (*ϕ_w_*).

Hexagonal boron nitride (h-BN) is a structural analogue of graphite with alternating boron (B) and nitrogen (N) atoms instead of C atoms, and unlike graphitic carbon, h-BN is electrically insulating but thermally conducting, which makes it especially suitable for microelectronic packaging applications [14,26,27]. Boron nitride nanotubes (BNNTs), on the other hand, are structurally similar to CNTs but have a wider temperature window for chemical inertness, high thermal conductivity, and electrically insulating behavior [27,28,29]. While the thermal enhancement of boron nitride (BN) derivatives has been investigated for different polymers including poly (vinyl butyral) [30], poly (siloxane) [31], poly (imide) [32], and epoxy resins [33,34,35], relatively fewer studies have focused on their impact on paraffins [14,36,37,38]. In particular, studies on the effect of BNNTs on paraffins are very limited [11]. Therefore, it is of interest to investigate the behavior of BN nanoadditives in paraffin materials for TES applications.

In this study, two different organic PCMs, n-nonadecane (C_19_H_40_, abbreviated as C_19_ henceforth) and n-eicosane (C_20_H_42_, abbreviated as C_20_ henceforth) were investigated. The choice of PCMs stemmed from their activity being close to summer temperatures (30–45 °C). To mitigate paraffin leakage, an epoxy resin was incorporated as the support matrix material, as reported in our previous studies [2]. A thickening agent, carbopol, was added to the epoxy–PCM formulations to minimize phase separation during sample synthesis. Nanostructured carbons including CNFs and CNTs, and BN particles and BNNTs were added as thermally conductive fillers in different gravimetric ratios, and their effect on thermophysical properties was analyzed. The formulations used three-dimensional (3D) printed and vibration-assisted printing (VAP) successfully, thereby demonstrating the feasibility of the direct material integration onto liners/containers for TES applications.

## 2. Results and Discussion

### 2.1. Microstructural Characterization

**Figure 1:**
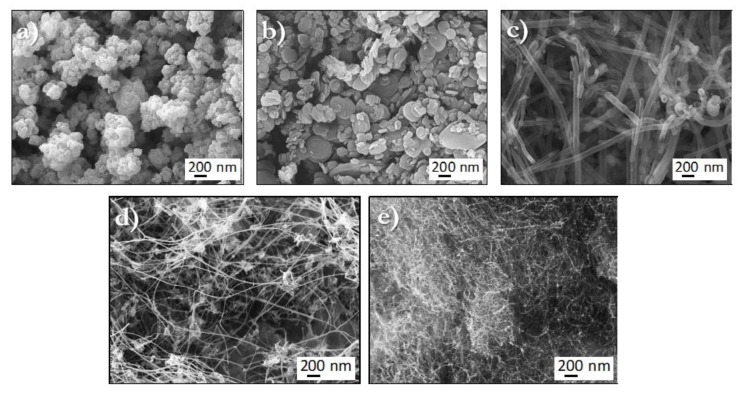
Representative SEM images of (**a**) carbopol; (**b**) boron nitride (BN) particles; (**c**) carbon nanofibers (CNFs); (**d**) boron nitride nanotubes (BNNTs), and (**e**) carbon nanotubes (CNTs) used for sample formulation.

Since the microstructure of the fillers can have a pronounced impact on the composite properties, morphological characterization was carried out using electron microscopy. The SEM micrographs of the starting powders are shown in Figure 1a–e. Carbopol (Figure 1a) comprised clusters of sub-micron scale particles. BN powders (Figure 1b) consisted of particles with a wide variation in size, ranging from 40 to 530 nm, with an average particle size of ~150 nm. The CNFs (Figure 1c) showed typical tubular structures with ~120-nm diameters. BNNTs (Figure 1d) and CNTs (Figure 1e) consisted of bundles of very thin tubular features. The diameter of the BNNTs ranged from 6 to 24 nm, whereas the diameter of the CNTs ranged from 4 to 15 nm.

### 2.2. DSC Analysis

**Figure 2:**
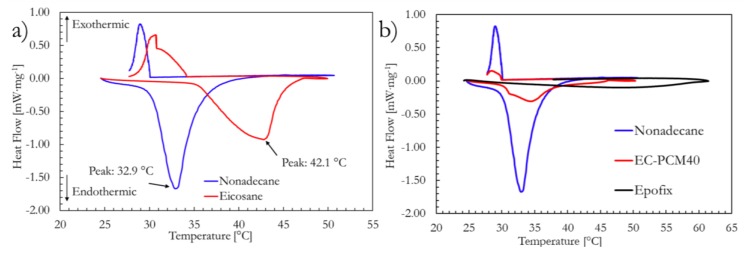
Differential scanning calorimetry (DSC) curves of the (**a**) two phase change materials (PCMs) (n-nonadecane and n-eicosane) and (**b**) PCM, epoxy, and the EC-PCM40 formulation.

To study the thermal characteristics of the samples, differential scanning calorimetry (DSC) analyses were carried out. Figure 2a shows the DSC thermograms of the two PCMs (n-nonadecane (C_19_) and n-eicosane (C_20_)) for comparison purposes. The thermograms were completely reversible for the employed testing conditions. In the endothermic segment, the transition/peak temperatures were noted as at 32.9 °C and 42.1 °C, and the corresponding latent heats were estimated as 160.2 J∙g^−1^ and 179.8 J∙g^−1^ for nonadecane and eicosane, respectively. The endothermic onset temperatures were noted as 29.7 °C and 35.2 °C for C_19_ and C_20_, respectively. Despite the higher latent heat exhibited by eicosane, nonadecane was chosen for further sample formulations owing to the proximity of its activity to ambient temperatures, which makes it more suitable for thermal regulation applications. Figure 2b shows the DSC thermograms of the nonadecane, bare epoxy (cured Epofix resin), and EC-PCM40 samples (sample composition details tabulated in Table 3). No endothermic or exothermic peaks were observed for the bare epoxy sample. The EC-PCM40 sample, on the other hand, exhibited two endothermic peaks—a small peak at 31.4 °C and a larger peak at 34.4 °C; the small peak could be a result of a solid–solid transition, whereas the pronounced peak is ascribed to the solid–liquid transition of the paraffin [7,39].

**Figure 3:**
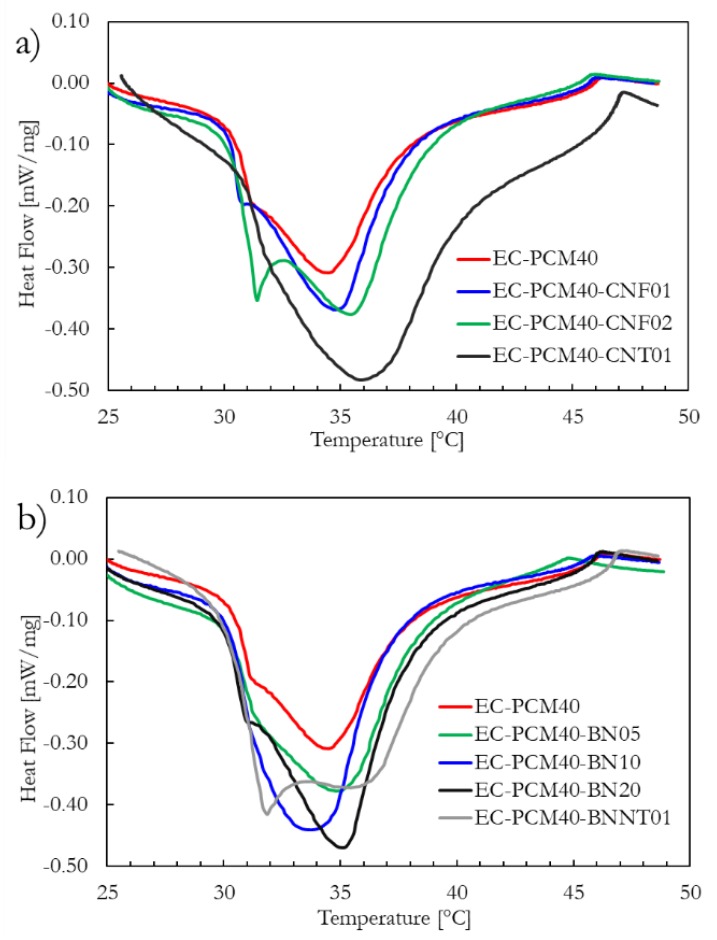
Heating curves of the (**a**) epoxy–PCM formulations incorporated with carbon nanofillers and (**b**) boron nitride and BNNT fillers.

Figure 3a,b shows the heating curves of the epoxy–carbopol–PCM formulations with nanostructured carbon fillers and BN fillers, respectively. The DSC analysis results of the sample formulations have been tabulated in Table 1. In Figure 3a, the small peak centered at ~31 ± 1 °C could be a result of solid–solid phase change, whereas the larger peak is attributed to the solid–liquid transition of the paraffin. The deviation in the transition temperature of the epoxy–PCM formulations with the additives could be a result of restrained paraffin molecule movement in the epoxy matrix; similar observations have been reported in other studies [6]. Interestingly, an enhancement in the latent heat is noted with the addition of CNF and CNT, as compared to the EC-PCM40 sample. However, the enhancement is much more significant in the EC-PCM40-CNT1 than in the EC-PCM40-CNF1 and EC-PCM-CNT2 composites. The improvement in the latent heat is ascribed to the intermolecular attraction between the nanostructured carbon and the paraffin [40]. Shaikh et al. modeled the change in latent heat in carbon nanoparticle-doped wax composites using an approximation for intermolecular attraction based on Lennard-Jones type potential and verified the theoretical predictions with empirical results; higher latent heat was reported for CNT/wax relative to CNF/wax, and the enhancement was attributed to the higher molecular density and the larger surface area of CNTs compared to the CNFs [40].

Similar to nanostructured carbon additives, BN-based epoxy–carbopol–PCM formulations (Figure 3b) also showed an increase in the latent heat with increasing BN content. The composite with 1 wt.% BNNT exhibited the highest latent heat among BN-based composites. However, the BNNTs in the EC-PCM40-BNNT01 sample aggregated and were hard to disperse homogeneously. Despite the higher latent heat exhibited by 1 wt.% CNT and 20 wt.% BN compositions relative to the 10 wt.% BN sample, the latter was chosen for 3D printing purposes due to the highly viscous nature of the pre-cured 1 wt.% CNT and 20 wt.% BN compositions, which made paste extrusion very challenging during the printing process.

**Figure 4:**
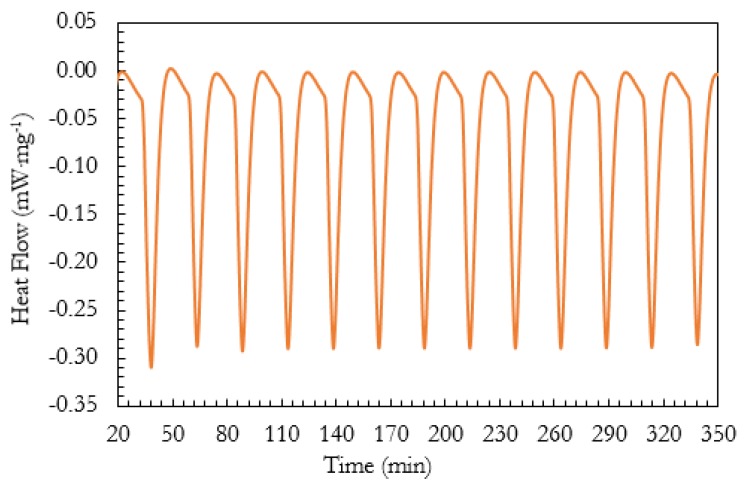
Heat flow over time for the EC-PCM40-BN10 sample for 12 cycles.

Figure 4 shows the heat flow in the EC-PCM40-BN10 sample for 12 cycles; after the first cycle, the sample showed excellent reversibility and repeatability during the thermal cycling.

### 2.3. Thermal Conductivity and Thermal Energy Storage Characteristics

**Figure 5:**
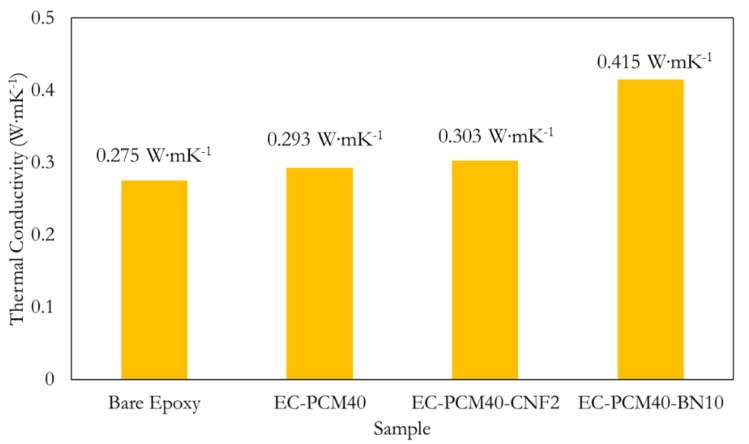
Thermal conductivities of the bare epoxy, EC-PCM40, EC-PCM40-CNF2, and EC-PCM40-BN10 samples.

To study the effect of thermal additives on the epoxy–paraffin samples, thermal conductivity (κ) measurements were carried out on the bare epoxy, EC-PCM40, EC-PCM40-CNF2, and EC-PCM40-BN10 samples, and the results are shown in Figure 5. The EC-PCM40-BN10 formulation showed the highest κ with a value of 0.415 W∙mK^−1^, followed by EC-PCM40-CNF2, EC-PCM40, and bare epoxy with values of 0.303 W∙mK^−1^, 0.293 W∙mK^−1^, and 0.273 W∙mK^−1^. Table 2 compares the thermal conductivity values of the sample formulations with other reports utilizing BN-based fillers. The values were comparable with other reports that indicated a similar gravimetric BN makeup.

**Figure 6:**
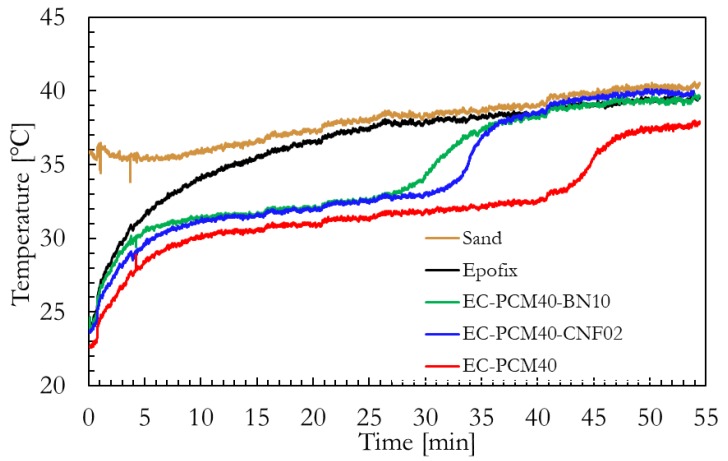
Temperature vs. time profiles of the epoxy–PCM–filler samples.

**Figure 7:**
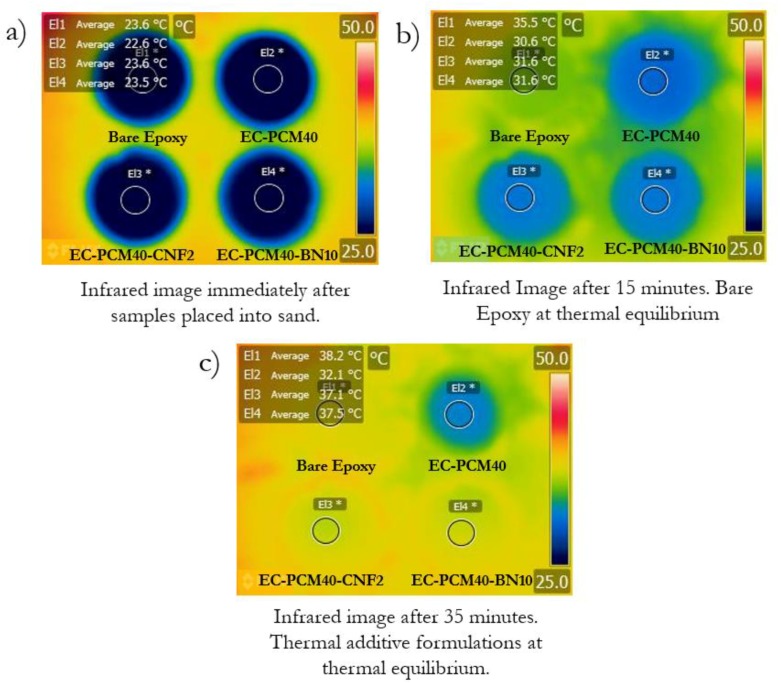
Infrared images of the bare epoxy, EC-PCM40, EC-PCM40-CNF2, and EC-PCM40-BN10 samples taken at different time intervals in the sand-bath experiment.

**Figure 8:**
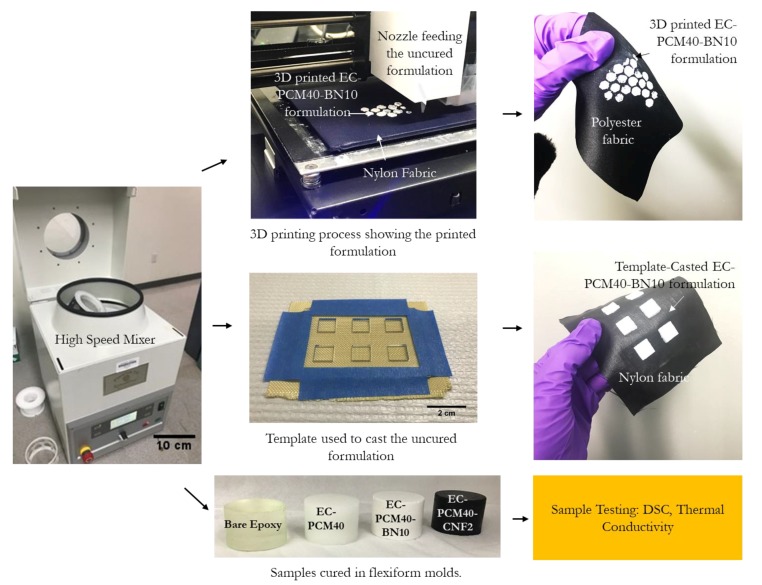
Images showing the asymmetric mixer, the three-dimensional (3D) printing setup, and printed samples on a polyester fabric, template used for casting and the casted samples on a nylon fabric, and samples cured in flexiform molds used for DSC and thermal conductivity testing.

Figure 6 shows the effect of enhanced thermal conductivity on the transition temperatures and heat storing times of the epoxy–PCM–filler samples. A qualitative comparison of the thermal energy storage characteristics of the formulations was made using the sand bath experiment shown in Figure 8. The epoxy–PCM–filler samples stayed ~5 °C cooler than the bare epoxy after the initial heat storage phase. The time taken by the EC-PCM40 sample to reach the transition temperature was higher than the EC-PCM40-CNF2 and EC-PCM40-BN10 samples. Furthermore, the length of the plateau for latent heat storage was also larger for the EC-PCM40 sample (~33 min) as compared to the samples with the thermal additives. The formulation with the 10 wt.% BN additive showed faster charging time than the 2 wt.% CNF composition, which can be explained by its higher thermal conductivity. It is noteworthy that during the experiment, the EC-PCM40 maintained lower temperatures than the formulations with thermal fillers for larger time periods. Figure 7 shows the representative thermal images captured by the infrared (IR) camera for the sample formulations at different time intervals: immediately after placing the samples into the sand bath (Figure 7a), when the bare epoxy reaches thermal equilibrium (Figure 7b), and when the thermal additive formulations are at thermal equilibrium (Figure 7c).

To summarize, enhancement in latent heat, thermal conductivity, and heat transfer was achieved with the addition of the thermal fillers. In addition to the improved thermophysical properties, the epoxy–carbopol–PCM composition with 10 wt.% BN showed excellent reversibility upon extended heating–cooling cycles. Furthermore, the formulations were successfully extruded/casted for 3D printing purposes, thereby demonstrating the feasibility of the direct integration of the material onto removable liners or portable containers for thermal regulation applications. Given that the PCMs solidify in exothermic conditions, the liners can be removed when the temperatures drop below the transition temperatures. Depending on the environment and temperatures, the presented method can be extended to other PCMs for TES purposes.

## 3. Materials and Methods

### 3.1. Precursor Materials

The epoxy resin system used to fabricate the composites was Epofix (Struers Inc. Cleveland, OH, USA). Epofix is a cold-setting resin based on two fluid epoxy components: Part A, which contains bisphenol-A diglycidylether, and Part B, containing triethylenetetramine, which functions as the hardener. The selection of the epoxy resin stemmed from the low viscosity and linear shrinkage expected of Epofix, since the preferred method to deposit the composite formulations would be a gel 3D printing system. Epofix cures in 8 to 24 h, has a viscosity of 550 cP (at 20 °C) and 150 cP (at 50 °C), and is resistant to acids, bases, acetone, and alcohol [41].

Carbopol (Sigma-Aldrich, St. Louis, Missouri, USA), a cross-linked polyacrylic acid polymer, was utilized as the thickening agent due to its known ability to stabilize and suspend pharmaceutical products. Experimental trials that attempted to use the epoxy resin and the PCMs without the thickener showed the separation of the phases, making the use of a thickening agent a necessity.

N-nonadecane and n-eicosane from Sigma-Aldrich (St. Louis, Missouri, USA) were used as the PCMs. The thermal additives selected were: i) carbon nanofibers (graphitized, platelets >98% carbon basis D × L 100 nm × 20 to 200 μm) at 1 wt.% or 2 wt.% loadings, ii) carbon nanotubes (multi-walled O.D. x L 6 to 9 nm × 5 μm, >95%) at 1 wt.% loading, and iii) boron nitride (~1 μm, 98%) particles at 5 wt.%, 10 wt.%, or 20 wt.% loadings, which were all from Sigma-Aldrich (St. Louis, Missouri, USA); and iv) boron nitride nanotubes at 1 wt. % loading from BNNT LLC (Newport News, Virginia, USA). The detailed sample composition containing component percentages has been tabulated in Table 3.

### 3.2. Fabrication of Epoxy–Carbopol–PCM Conducting Agent Composites

The PCM was melted using a water bath and then mixed with Part A using a dual asymmetric speed mixer (Flacktek, Landrum, SC, USA). The thickening agent was added followed by the other fillers (CNF, CNT, BN, or BNNT). Part B of the epoxy system was finally added to the mixture, and the formulation was left to cure at room temperature in flexiform molds (Struers, Inc. Cleveland, OH, USA). The process flow representative samples are shown in Figure 8.

### 3.3. Characterization and Testing

**Figure 9:**
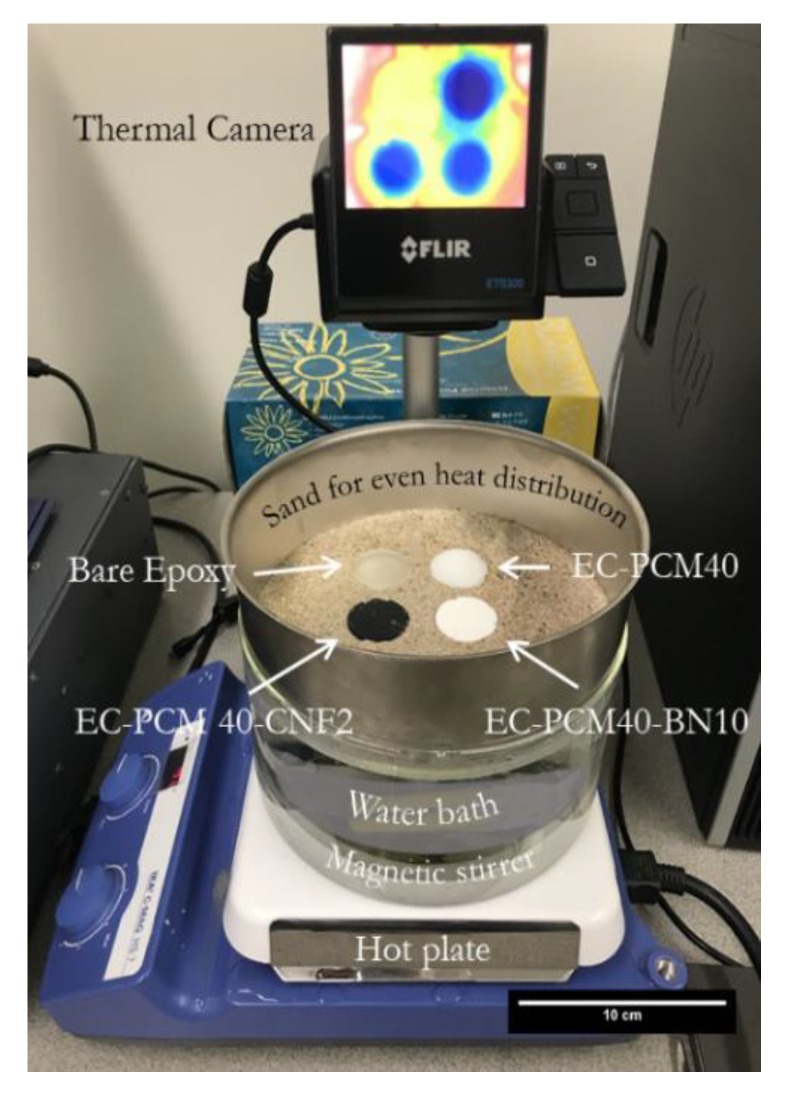
A photograph showing the sand-bath experimental setup used to measure the temperature vs. time profiles of the bare epoxy, EC-PCM40, EC-PCM40-CNF2, and EC-PCM40-BN10 samples.

Microstructural characterization on the starting powders (carbopol, BN, CNF, BNNT, and CNT) was carried out using a scanning electron microscope (Zeiss Neon 40 FE-SEM) in the secondary electron (SE) mode. Differential scanning calorimetry (DSC) measurements on the samples were performed using a Netzsch STA 449 F3 Jupiter Simultaneous Thermogravimetric Analysis (STA) instrument that was capable of the simultaneous acquisition of both the DSC and thermogravimetric analysis (TGA) data. The DSC experiments were performed in synthetic air (80:20 N_2_/O_2_ *v:v*). As a simple proof-of-concept, the temperature versus time profiles of the samples were studied using a sand bath setup and a FLIR ETS320 infrared camera (shown in Figure 9); sand was used to ensure even heat distribution. Thermal conductivity measurements were carried out using a C-Therm TCi Thermal Conductivity Analyzer using the modified transient plane source (MTPS) configuration. Thermal conductivity measurements were performed at room temperature, and the service was contracted with Thermal Analysis Labs (Fredericton, N.B., Canada).

### 3.4. D Printing Conditions

The EC-PCM40-BN10 samples were 3D printed using vibration-assisted printing (VAP) [40]. VAP uses resonant nozzle vibrations in a direct-write system to reduce effective friction at the nozzle exit, and it has been used for dispensing highly viscous mixtures [42] with high solids loadings reaching 76 vol.% [43]. The uncured formulation was loaded into a 6-mL polypropylene syringe with a nozzle diameter of 0.6 mm and printed into the desired pattern with a thickness of 0.4 mm and a radius of 5 mm on the substrate fabric at a print rate of 20 mm s^−1^. A vibration amplitude of 8 µm and a back pressure of 25 psi was used for the VAP system. The vibrations increased the temperature at the nozzle to a range of 28 to 32 °C, which locally melted the paraffin and acted synergistically with the friction reduction to induce flow in a controlled way.

### 3.5. Template Casting

The uncured EC-PCM40-BN10 formulation was casted onto the fabric substrate using a six-square patterned acrylic template. The formulation was poured through the template, after which the template was removed, and the sample was allowed to cure overnight.

## 4. Conclusions

In this study, n-nonadecane (C_19_H_40_) was studied as the PCM material, owing to its thermal activity in desired temperature ranges (30 to 42 °C). To mitigate the paraffin leakage, an epoxy resin was incorporated as the support matrix material, and carbopol ensured minimal phase separation during sample synthesis. Nanostructured carbons including CNFs and CNTs, BN particles, or BNNTs were added as conductive fillers in different gravimetric ratios to improve the inherently low thermal conductivity of paraffins. Enhancement in thermophysical properties including latent heat, thermal conductivity, and heat transfer was achieved with the addition of the thermal fillers. In addition to the improved thermal characteristics, the epoxy–carbopol–PCM composition with 10 wt.% BN showed excellent reversibility upon extended heating–cooling cycles. Furthermore, the sample was successfully extruded on polyester/nylon fabrics using vibration-assisted 3D printing, thereby demonstrating the feasibility of the direct integration of the material onto removable liners or portable containers of different geometries for thermal regulation. The presented method is anticipated to apply to other PCMs for TES applications.

## Figures and Tables

**Table 1:** DSC analysis results for different sample compositions.

Sample	Latent Heat (J∙g^−1^)	Transition Temperature (°C)
Nonadecane (C-19)	160.2	32.9
EC-PCM40	27.2	34.4
EC-PCM40-CNF01	27.1	34.6
EC-PCM40-CNF02	39.6	35.4
EC-PCM40-CNT01	74.6	35.8
EC-PCM40-BN05	32.2	34.8
EC-PCM40-BN10	41.6	33.7
EC-PCM40-BN20	42.6	35.1
EC-PCM40-BNNT01	59.5	35.5

**Table 2:** Thermal conductivity comparison with other reports. h-BN: hexagonal boron nitride.

Matrix Material	Filler	Filler Fraction	Thermal Conductivity	Ref
Paraffin	h-BN	10 wt.%	~0.2 W∙mK^−1^	14
Paraffin	Porous h-BN scaffolds prepared by dispersion in polyvinylpyrrolidone (PVP), Na_2_SiO_3_, followed by freeze drying and calcination	10 wt.%	0.45 W∙mK^−1^	14
Poly (vinyl butyral) (PVB)	BNNT	18 wt.%	~1.81 W∙mK^−1^	30
Poly (siloxane)	BN	30 vol.%	~0.28 W∙mK^−1^	31
Poly (imide)	BN micro and nanopowders	10 wt.%	~0.26 W∙mK^−1^	32
Poly (imide)	BN micro and nanopowders	30 wt.%	~1.2 W∙mK^−1^	32
Paraffin	h-BN nanosheets	10 wt.%	~0.48 W∙mK^−1^	37
Paraffin–epoxy	BN particles	10 wt.%	~0.415 W∙mK^−1^	This work

**Table 3:** Sample composition details.

Sample	Filler	Epoxy (Resin + Hardener) (wt.%)	Carbopol (wt.%)	PCM (n-nonadecane) (wt.%)	Filler (wt.%)
Bare epoxy	None	100	0	0	0
EC-PCM40	None	55	5	40	0
EC-PCM40-CNF01	CNF	54	5	40	1
EC-PCM40-CNF02	CNF	53	5	40	2
EC-PCM40-CNT01	CNT	54	5	40	1
EC-PCM40-BN05	BN	50	5	40	5
EC-PCM40-BN10	BN	45	5	40	10
EC-PCM40-BN20	BN	35	5	40	20
EC-PCM40-BNNT01	BNNT	54	5	40	1
